# Verapamil-Induced Hypotension in End-Stage Renal Disease: The Role of Calcium Gluconate

**DOI:** 10.7759/cureus.33341

**Published:** 2023-01-04

**Authors:** Neerusha Kaisbain, Wei Juan Lim, Vareeja Kaisbain

**Affiliations:** 1 Cardiology, Hospital Queen Elizabeth II, Sabah, MYS; 2 Cardiology, National Heart Institute (IJN), Kuala Lumpur, MYS; 3 Internal Medicine, Sultan Ismail, Johor Bahru, MYS

**Keywords:** tachyarrhythmia, supraventricular tachycardia (svt), hypotension, calcium gluconate, verapamil

## Abstract

Verapamil is a useful drug in supraventricular tachycardias, atrial flutters, and fibrillations. However, its usage is accompanied by an undesirable side effect of hypotension. This limits its usage in patients where even the slightest reduction of blood pressure for a brief period may prove detrimental, e.g., in patients with critical coronary artery disease. Intravenous calcium given as pretreatment to verapamil prevented verapamil-induced hypotension. Its usage after hypotension restored the blood pressure to its baseline level. All these occur without the loss of the antiarrhythmic effect of verapamil. Furthermore, the pharmacokinetics of verapamil is unaltered in patients with chronic kidney disease. Thus, no dosage adjustment is required in this population. Here we describe a case of verapamil-induced hypotension in a patient with end-stage renal failure, which was reverted with intravenous calcium administration without altering the atrioventricular blockade effect of verapamil.

## Introduction

Chronic kidney disease causes complex alterations in metabolic homeostasis, autonomic nervous system, and cardiovascular system, predisposing them to various arrhythmias [1]. A reduction in renal clearance of drugs, on the other hand, makes patients with chronic kidney disease more prone to the accumulation of antiarrhythmic drugs, predisposing them to its adverse effect [1]. Verapamil is an effective antiarrhythmic agent used in patients with chronic kidney disease. Its pharmacokinetics is unaltered in this population [2]. Unfortunately, the use of verapamil in tachyarrhythmias is hampered by its side effect of hypotension, especially in those with poor left ventricular dysfunction [3,4]. The administration of calcium gluconate before verapamil blunted the hypotensive effect of verapamil. In cases where verapamil-induced hypotension occurs, calcium gluconate helps return the systolic blood pressure to its baseline value. The beneficial effect of calcium gluconate occurs without diminishing the beneficial antiarrhythmic effect of verapamil [3-5].

## Case presentation

**Figure 1:**
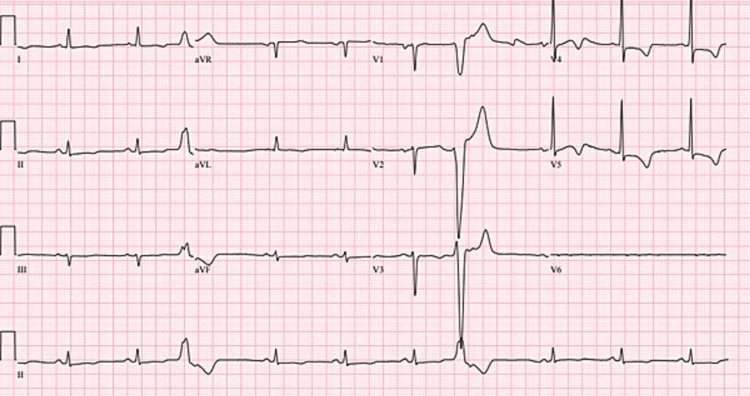
ECG showing sinus bradycardia with occasional premature ventricular contractions.

A lady in her mid 60s, previously diagnosed with diabetes mellitus, hypertension, and chronic kidney disease, presented with progressively worsening body weakness of one-month duration with loss of appetite and dyspnoea on exertion. She was under nephrologist consultation for her chronic kidney disease. However, she defaulted on her appointments. Upon admission, she was in end-stage renal failure, with a serum potassium of 6.9mmol/L, pH of 7.19, bicarbonate level of 9.3mmol/L, urea level of 46mmol/L, creatinine level of 1555 micromol/L, and eGFR of 2ml/min/1.73m2. She also had an acute hepatocellular injury with alanine transaminase of 856U/L and aspartate transaminase of 385U/L. Total bilirubin and alkaline phosphatase were within normal limits. Her hemoglobin level was 5.5g/dL. ECG on admission showed sinus tachycardia (Figure 1). The chest radiograph showed normal heart size. She was diagnosed with the end-stage renal disease with hyperkalemia, metabolic acidosis, renal anemia, and acute hepatocellular injury. A dialysis catheter was inserted over the patient's femoral vein, and she was commenced on urgent hemodialysis. She was transfused with 1 pint of the packed cell.

**Figure 2:**
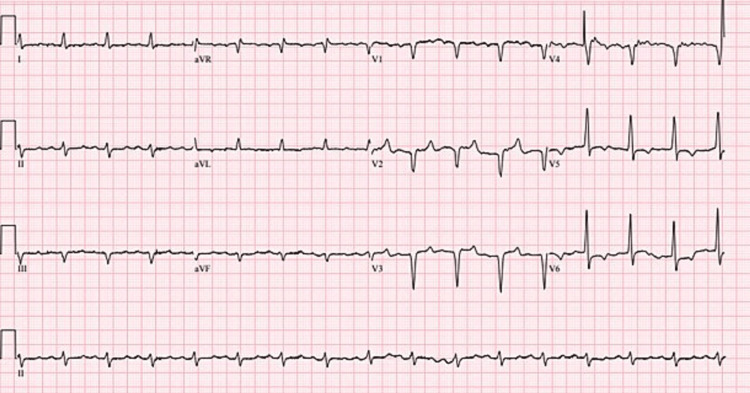
ECG on admission showed sinus tachycardia.

**Figure 3:**
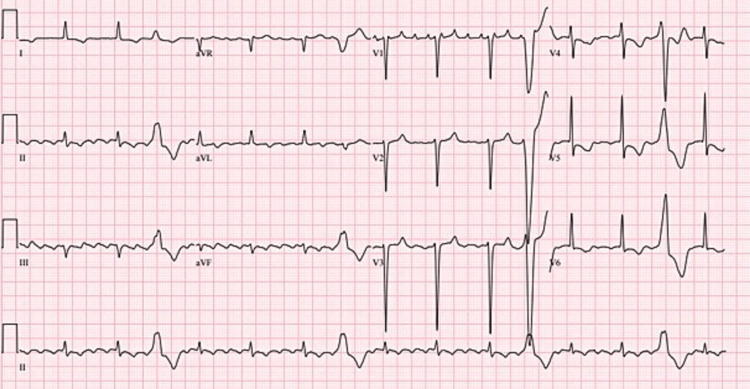
ECG showing atrial flutter with 4:1 with occasional premature ventricular contractions.

Upon completing her hemodialysis, the patient's heart rate was 147 beats per minute. Her blood pressure was normal at 147/82mmHg with an oxygen saturation of 95% under room air. The cardiac monitor showed regular narrow complex tachycardia. The carotid massage was attempted but to no avail. An ECG was performed, and it revealed atrial flutter of rate 150 with 2:1 conduction (Figure 2). The patient was asymptomatic. IV adenosine was initially administered to induce a conduction block over the AV node to diagnose the underlying rhythm correctly. Flutter waves were revealed on the cardiac monitor shortly after the administration of adenosine, but it did not abort the tachyarrhythmia. As the patient had liver and kidney impairment, a decision was made against using digoxin and amiodarone. Unfortunately, an echocardiogram was not readily available in the district hospital. Considering that the chest radiograph demonstrated normal heart size and the patient had no previous history of cardiac failure, an intravenous verapamil 5mg infusion was given over five minutes instead. The atrial flutter rate was reduced to 95 per min with a BP of 124/82mmHg and sPO2 of 99% under nasal prong oxygen 3L/min. A systolic blood pressure reduction of 23mmHg was observed. ECG showed atrial flutter with 4:1 with occasional premature ventricular contractions (Figure 3).

**Figure 4:**
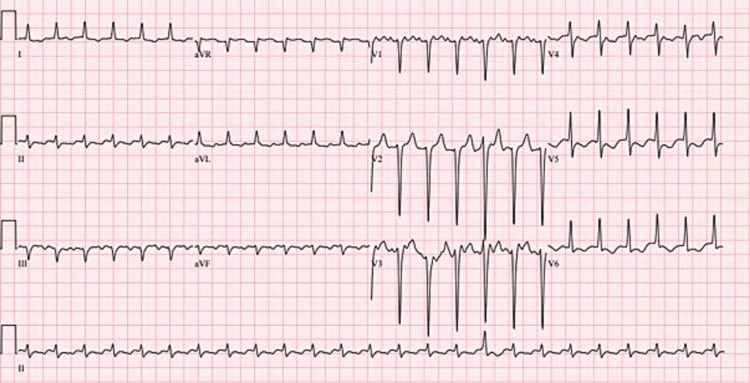
ECG showing atrial flutter of rate 150 with 2:1 conduction.

**Figure 5:**
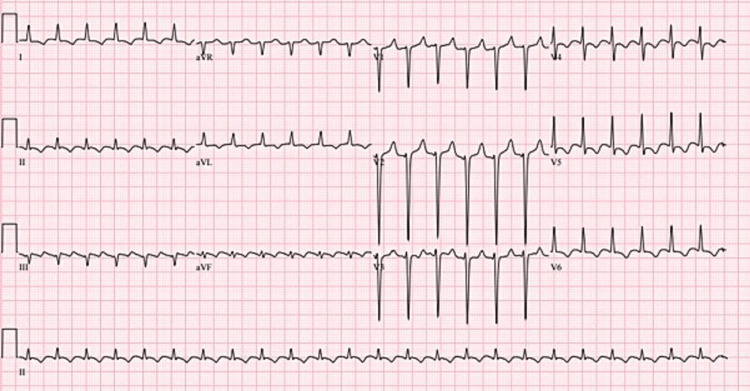
ECG showing atrial flutter with 2:1 conduction.

**Figure 6:**
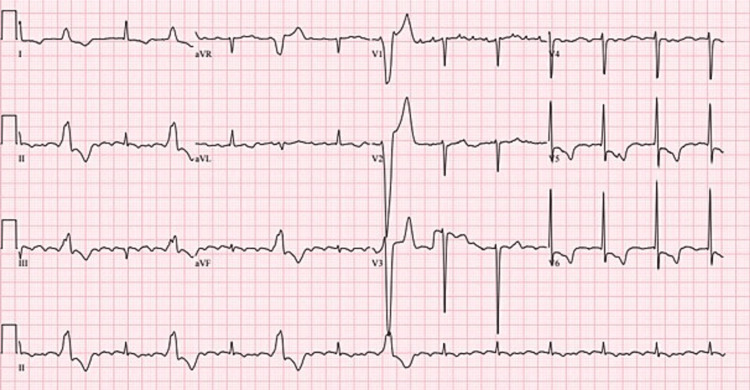
ECG shows atrial flutter with 4:1 conduction and occasional premature ventricular contractions.

Later that night, a continuous cardiac monitor showed atrial flutter with a rate of 145/min. The patient remained asymptomatic. BP was 109/70. An electrocardiogram showed atrial flutter with 2:1 conduction (Figure 4). Another IV Verapamil 5mg slow infusion was given over five minutes. Repeated ECG showed atrial flutter with 4:1 conduction and occasional premature ventricular contractions (Figure 5). However, the blood pressure dropped to 74/50mmHg. This time, a reduction of systolic blood pressure by 35mmHg and diastolic blood pressure of 20mmHg was observed. A trial of 50ml of normal saline bolus over 30 minutes was given. However, the blood pressure remained low at 80/50mmHg. IV calcium gluconate 10% 10mls was given over 10 minutes, containing 940 mg of calcium gluconate, essentially 90mg of elemental calcium. The patient's blood pressure increased to 103/70mmHg a few minutes after administration and remained stable afterward. An ECG performed the following day showed sinus bradycardia with occasional premature ventricular contractions (Figure 6).

Table 1 shows her serial blood investigations. She had severe metabolic acidosis and hyperkalemia with end-stage renal failure and severe anemia on admission. These quickly resolved after the hemodialysis. Her thyroid function test was normal. Ultrasound of her abdomen showed a normal liver with the bilateral renal parenchymal disease. One week after the cardiac event, an echocardiogram showed poor left ventricular function with an ejection fraction of 22%, moderate mitral regurgitation, dilated left atrium, and global hypokinesia. The patient underwent esophagogastroduodenoscopy (OGDS) due to low hemoglobin, revealing Forrest IIc ulcers over the gastric antrum and duodenum. It was treated with an adrenaline injection.

**Table 1:** Serial blood investigations.

	Admission	After Dialysis	Day 2
Hemoglobin (g/dL)	5.5	7.7	8.1
Sodium (mmol/L)	140	137	135
Potassium (mmol/L)	6.9	4.7	4.8
Urea (mmol/L)	46	23	26
Creatinine (umol/L)	1555	876	931
Potential of hydrogen (pH)	7.19	7.20	7.38
Bicarbonate (mmol/L)	9.3	10.2	17.2
Bilirubin (umol/L)	16.3	14	8
Albumin (g/L)	30	31	30
Aspartate transaminase (u/L)	385	237	179
Alanine transaminase (u/L)	856	755	632
Alkaline phosphatase (u/L)	147	137	120
Corrected calcium (mmol/L)	2.2		2.1
Thyroid-stimulating hormone (mU/L)		1.08	
Thyroxine (pmol/L)		14.2	

The patient received regular hemodialysis throughout her hospitalization. She was subsequently transferred to a tertiary center for nephrologist consultation and insertion of a Tenckhoff catheter for continuous ambulatory peritoneal dialysis. Anticoagulant was not commenced because of the high bleeding characteristic of the patient. She was discharged with a beta-blocker and referred to the cardiology service to consider atrial flutter ablation later.

The patient underwent Tenckhoff catheter insertion in the tertiary center and was initiated on continuous ambulatory peritoneal dialysis (CAPD). 

## Discussion

Verapamil is a non-dihydropyridine calcium channel blocker. It can be used to treat hypertension, supraventricular arrhythmias, and angina. It is a class IV antiarrhythmic drug effective against supraventricular tachyarrhythmias (SVT) [3]. Verapamil exerts its antiarrhythmic effect by increasing the atrioventricular (AV) nodal refractory period and disrupts AV nodal conduction to His bundle [3]. It slows down the ventricular rate in atrial flutters or fibrillations within minutes and converts paroxysmal SVTs to sinus rhythms [3,4]. It is an alternative drug for SVTs that are unresponsive or contraindicated to adenosine [4]. Its use is contraindicated in those with accessory bypass tracts, such as Wolff Parkinson White syndrome (WPW) or Lown-Ganong-Levine (LGL) syndromes, as increasing the refractory period in the AV node in such patients encourages the antegrade conduction through the accessory pathway causing life-threatening rapid ventricular rate in atrial flutter or fibrillation [6,7].

Verapamil can be used in patients with chronic kidney disease. In fact, verapamil has a vasodilatory effect on the renal arteries, causing an improvement in renal perfusion and ultimately protecting the kidney and improving its function [8]. The pharmacokinetics of verapamil is unaltered in patients with chronic kidney disease, and additional verapamil dosage is not required in end-stage renal disease patients post hemodialysis [2].

Verapamil acts on myocardial cells and vascular smooth muscles. The most common side effect of verapamil is hypotension [4]. As a calcium channel blocker, it blocks the entry of calcium into the myocardial cells through slow channels during depolarization, causing a decrease in myocardial contractility and resulting in a negative inotropic effect [3]. It also reduces vascular smooth muscle tone, causing peripheral vasodilatation and ultimately reducing blood pressure [3]. Decreased calcium in contractile proteins in the heart and smooth muscle leads to these effects [4]. Up to 75% of patients treated with intravenous verapamil experienced a reduction in blood pressure for up to 25 minutes after administration, with 25% experiencing systolic blood pressure (SBP) of less than 100mmHg [4]. Verapamil reduces systolic blood pressure by approximately 5 - 40mmHg, while diltiazem, the other non-dihydropyridine calcium channel blocker, shows a similar mean reduction in SBP [4]. The usual dose of intravenous verapamil is 5 - 20mg. The degree of SBP reduction appears to be dose-related. A higher dose of verapamil results in a greater reduction in myocardial contractility and, thus, the blood pressure [3]. Some patients are more vulnerable to verapamil-induced hypotension, including the elderly population, those with concomitant beta-blocker usage, significant left ventricular dysfunction, and those with low baseline blood pressure [4]. In fact, administering verapamil in patients with critical coronary artery disease may be harmful as even transient mild hypotension in these patients may prove fatal [3].

The negative inotropic effect of verapamil limits its use in patients with poor left ventricular function [3]. It is safe in patients with normal or mildly reduced left ventricular function, as the negative inotropic effect of verapamil and its peripheral vasodilatory effect are counterbalanced [9]. Its use in patients with severe left ventricular dysfunction, however may precipitate life-threatening acute decompensation of cardiac failure, causing pulmonary oedema and cardiogenic shock [9]. Hence, its use in patients with severe congestive heart failure is contraindicated [6].

In SVTs, atrial fibrillations, or flutters, a persistently rapid heart rate can have adverse hemodynamic effects, leading to morbidity or even fatality [5]. Administration of intravenous verapamil reduces the mean ventricular heart rate by 21%, and some patients were restored to sinus rhythms at the peak effect of verapamil [5]. The effective reduction in heart rate was accompanied by the undesirable side effect of hypotension. Studies found that intravenous calcium gluconate administration immediately after verapamil restored blood pressure in these patients [3-5]. A pretreatment with calcium gluconate before verapamil administration prevented verapamil-induced hypotension [3-5]. What is intriguing is that the administration of calcium gluconate did not impair the antiarrhythmic effect of verapamil. It is shown that there are no statistically significant differences in heart rate reduction due to verapamil in patients who received and did not receive calcium pretreatment [5]. In essence, intravenous calcium gluconate administration corrected and even prevented undesirable side effects of hypotension as a result of verapamil without altering its desirable antiarrhythmic properties [3,5]. Intravenous calcium can be considered an antidote to verapamil-induced hypotension [3]. Calcium is used in patients with verapamil overdose, although the response is extremely variable [10].

In vivo studies in animals showed that calcium completely reverses the reduction in cardiac output and myocardial contractility due to verapamil [4]. It partially reverses the verapamil's depressant effect on systolic and diastolic blood pressures. However, it does not affect verapamil-induced changes in AH interval, which is the conduction time from low right atrial tissue through the AV node to the bundle of His [4]. These studies show the effects of calcium on electrophysiologic and hemodynamic effects of verapamil are distinct [4]. Weiss et al. proposed that the transient increase in the plasma calcium level reversed the reduction of calcium influx into the myocardium and vascular smooth muscle triggered by verapamil but not in cardiac conducting tissues [3]. Perhaps, cardiac conducting tissues are less sensitive to changes in extracellular calcium concentration, and a much higher dose of calcium is required to reverse the AV nodal delay produced by verapamil [3]. One may administer intravenous calcium before verapamil infusion as prophylaxis against verapamil-induced hypotension, especially in those where even transient hypotension may have detrimental effects [4]. A dose of 9 to 270mg of elemental calcium administered over 1 to 8 minutes might help prevent verapamil-induced hypotension [4]. Rapid administration of intravenous calcium may cause flushing, ventricular fibrillation and severe bradyarrhythmias [6].

Our patient experienced stable atrial flutter, which was probably precipitated by many factors. She had severe hyperkalemia, metabolic acidosis, and anemia due to renal failure in the background of an undiagnosed left ventricular dysfunction. In addition, the commencement of hemodialysis might have stressed the heart further, precipitating tachyarrhythmia. The presence of liver injury and renal injury served as a relative contraindication in using the usual antiarrhythmic drugs, such as digoxin and amiodarone. The patient did not respond to adenosine either. Thus, verapamil was infused. It successfully reduced the ventricular rate. However, the patient experienced verapamil-induced hypotension despite the minimal dose of 5mg. In the first instance, there was a reduction of systolic blood pressure by 23mmHg. A drop of 35mmHg of systolic blood pressure was observed in the second instance, but with a much lower baseline heart rate. This corresponds to the clinical trials stating that verapamil causes a mean reduction in systolic blood pressure by 5 to 40mmHg. However, the hypotensive effect lasted longer, i.e., more than 30 minutes. The patient is predisposed to verapamil-induced hypotension due to the presence of left ventricular dysfunction and a low baseline blood pressure, i.e., 109/70mmHg. She received intravenous calcium gluconate slow infusion afterward, which restored her blood pressure to her baseline level without causing a rapid ventricular response of the atrial flutter.

## Conclusions

Pretreatment with intravenous calcium blunted or even prevented verapamil-induced hypotension. If given after verapamil, calcium restored the blood pressure to control values. Either way, calcium did not dampen the desirable antiarrhythmic effect of verapamil. This makes calcium gluconate a considerable option in patients with verapamil-responsive supraventricular arrhythmias but poor tolerance to the slightest drop in blood pressure.
